# 78696 A Qualitative Cross-Sectional Study of Leadership in a Pandemic: What do Students Value?

**DOI:** 10.1017/cts.2021.568

**Published:** 2021-03-30

**Authors:** Alec Bernard, Sarah Contreras-Ortiz, Elizabeth Jones, Michael Heung, Timothy C. Guetterman, Nell Kirst

**Affiliations:** University of Michigan Medical School

## Abstract

ABSTRACT IMPACT: This real-world study of what students value in crisis leadership fills an important gap in the literature and may inform future leadership development programs in undergraduate medical education. OBJECTIVES/GOALS: Leadership training is of growing importance and prevalence in medical education. The COVID-19 pandemic provides a unique insight into the qualities students value in leaders. Our qualitative study examined these leadership themes and provides a grounding for future development of leadership programs. METHODS/STUDY POPULATION: A conventional qualitative approach was used in order to allow open expression of ideas related to leadership in a pandemic. The authors developed a 5 free-text question survey instrument aimed to uncover student perceptions of leadership both during the current pandemic and in crises in general. A participant pilot was performed in order to ensure readability and ease of understanding. We used thematic analysis to examine the content of the survey responses, and inductive coding of the responses allowed identification of emerging themes. Medical students at the University of Michigan were surveyed. RESULTS/ANTICIPATED RESULTS: In total, 162 students completed the survey. The demographic characteristics of participants are shown in Table 1. Median age was 25 years old (range, 22-39). There was good representation from the 4 classes in the medical school with 20-30% from each medical school class and 5% of dual degree students. Thematic analysis demonstrated that students value personal characteristics of excellence in their leaders with an orientation towards helping other people. Students believe that leaders must know how to interpret and use information and then that these leaders must be able to communicate expertly to guide organizations. The final theme that emerged is that effective leaders must commit to decisive action. DISCUSSION/SIGNIFICANCE OF FINDINGS: This study took place at a time of unprecedented crises and response examples were grounded in this real-world practice of leadership. These results and themes that emerged fill a critical gap and may facilitate future curriculum development for medical students and trainees.

